# The results of subthreshold 577 nm yellow laser application using pascal laser system in patients with chronic central serous chorioretinopathy

**DOI:** 10.1007/s10103-025-04522-8

**Published:** 2025-06-09

**Authors:** Mehmet Önen, Kürşad Ramazan Zor, Levent Doğan, Erkut Küçük, Gamze Yıldırım Biçer, Ömer Özer

**Affiliations:** 1 Ankara City Hospital, Çankaya, Turkey; 2 Niğde Ömer Halisdemir University, Niğde, Turkey

**Keywords:** Central serous retinopathy, Subthreshold laser, Subretinal fluid, Retina pigment epithelial changes

## Abstract

To report the results of Endpoint Management (EpM) with PASCAL laser in the treatment of chronic central serous retinopathy (CSR). A 577 nm yellow laser was applied using PASCAL with EpM to 11 eyes on 11 patients who were followed for CSR for at least 12 months in this prospective study. The laser spot size was set to 200 μm, the pulse duration was 15 msec, and the spot spacing was set to 0.25 diameter. In cases where recurrence was present or subretinal fluid did not disappear, the laser was applied again three months after the last laser treatment. Detailed anterior segment and fundus examination, intraocular pressure measurement, and optical coherence tomography (OCT) were performed. Fundus fluorescein angiography (FFA) was performed at the time of diagnosis and at the end of the 6th month. The treatment was applied once for four patients (38.3%), two times for five patients (45.4%), three times for one patient (9.1%), and five times for one patient (9.1%) to achieve these results. The average pre-laser central macular thickness (CMT) value was 343.3 ± 50.2 μm, and post-treatment, the CMT value decreased to 233.1 ± 20.3 μm (*p* = 0.008). According to baseline examinations, the patients’ mean pre-treatment BCVA (logMAR) value was 0.46 ± 0.18, which improved to 0.34 ± 0.31 with treatment (*p* = 0.012). Five patients (45.4%) had retinal pigment epithelial changes, and no other patients had complications. PASCAL with EpM using a 577 nm yellow laser is an effective and reliable treatment for patients with chronic CSR. The frequency of retinal pigment epithelial change was higher than in other studies in the literature in our study.

## Introductıon

Central serous chorioretinopathy (CSR) is a retinal disorder characterized by idiopathic serous detachment of the neurosensory retina in the macula and may accompany focal detachment of the retinal pigment epithelium (RPE) [1]. Although etiology is unclear and many factors have been implicated, the basic pathophysiology is thought to be hyperpermeability of the choroidal vessels, impaired choroidal vascular autoregulation, impaired pump function, and the barrier function of RPE [2]. CSR is mostly self-limiting, and there is no significant vision loss in patients in the first episode. However, recurrence rates up to 50% have been reported in literature [3]. Unlike acute cases, recurrence and chronic cases may result in progressive retinal atrophy and severe permanent vision loss; therefore, these cases need to be treated [4]. Conventional focal laser, photodynamic therapy (PDT), intravitreal anti-vascular endothelial growth factor (anti-VEGF) agents, transpupillar thermotherapy, subthreshold micropulse yellow laser, and subthreshold micropulse diode laser have been the main treatment methods used so far [4].

PDT, which is an effective treatment method in CSR, can cause important complications such as atrophy in the retinal pigment epithelium, ischemia in the choriocapillaris, choroidal neovascularization, and reactivate RPE hyperplasia. Moreover, PDT is a more invasive method compared to laser treatment methods, and an intravenous drug infusion, which is quite expensive, is applied. The patient should stay away from sun exposure for a few days after treatment [5–7]. Recently, using half-dose PDT, the PLACE study demonstrated favorable results in CSR patients, but PDT laser system is not common in most clinical settings.

Conventional focal laser treatment may cause central and paracentral scotomas, loss of contrast sensitivity, accidental foveal damage, choroidal neovascularization, and retinal distortion. These possible complications limit the use of conventional lasers in the treatment of CSR, especially in subfoveal and juxtafoveal leaks [3]. In order to overcome these possible complications and limitations, there has been a need to modify the laser parameters, power, pulse duration, and repetition [8]. Various modifications have been tried by physicians, and all of these have been collected under the title of nondamaging retinal laser. The laser power is set to be subthreshold in the treatments. Threshold lasers have a barely visible effect on the retina [9]. 810 nm diode laser and 577 nm yellow laser are the most frequently used lasers in literature in subthreshold micropulse laser applications. Until recently, however, there was no standardized procedure for the subthreshold laser, which was reported to be effective and did not cause chorioretinal damage in the treatment of CSR. EndPoint Management (EpM) is another non-damaging retinal laser therapy algorithm, and it is available on the PASCAL laser device (Topcon Medical Laser Systems, Santa Clara, CA) [10, 11]. Pascal laser uses a yellow laser at a wavelength of 577 nm. Pascal laser regulates laser power and pulse duration with EpM software, which allows the application of 30% of the power of the threshold laser in a 15-ms pulse duration [10, 11]. In this study, we aimed to report our results of PASCAL laser with EpM in the treatment of chronic CSR patients.

### Matherıal and methods

This study was conducted prospectively between January 2022 and January 2023 at the Ophthalmology Department. This study received approval from the local Ethics Committee (2019/12 − 07). All procedures adhered to the tenets of the Declaration of Helsinki, and detailed information about the procedures was given to all patients involved in the study and written and verbal consent was obtained from all patients.

**Fig. 1:**
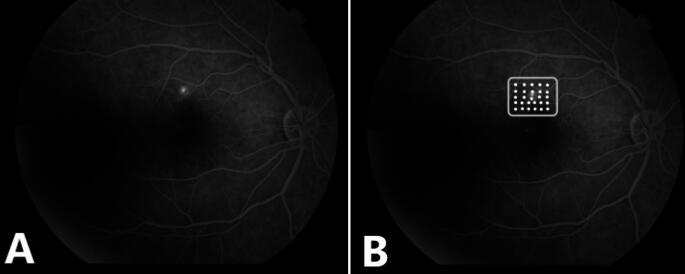
In the fundus fluorescein angiography, the leakage zone (**A**), the area delineated by the rectangle represents the laser-treated region (**B**)

Patients with CSR and symptoms lasting for at least four months who did not receive treatment other than nepafenac eye drops during this period were included in the study. In all patients, the best corrected visual acuity (BCVA) was examined using an early treatment for diabetic retinopathy study chart and the logMAR scale was used for analysis. A detailed anterior segment and fundus examination were performed, and intraocular pressure was measured using a Goldman applanation tonometer. Serous macular detachment was detected using optical coherence tomography (Cirrus HD-OCT, Carl Zeiss Meditec, Germany), and focal or diffuse RPE leakage was detected using Fundus fluorescein angiography (FFA) (Fig. 1).

Exclusion criteria were diabetic retinopathy, hypertensive retinopathy, hereditary retinopathy, epiretinal membrane, chorioretinitis, retinal or choroidal disease other than CSR or previous retinal or macular laser, previous intravitreal steroid or anti VEGF applications, conditions like cataract that cause decrease in vision and / or prevent the acquisition of fundus image.

In the first 6 months, detailed eye examinations, BCVA, and OCT imaging were performed monthly. The FFA was performed again in the sixth month. PASCAL laser (Topcon Medical Laser Systems, Santa Clara, CA) was applied to and around the area where fluorescein leakage was detected in FFA and macular thickening in OCT. Volk Area Centralis (Volk Optical Inc., Mentor, Ohio, USA) contact lens was used during the application. Firstly, the laser power that produced mild retinal whitening in the superonasal retina was determined by the physician, and this was accepted as threshold power. PASCAL laser with EpM adjusts the laser power to 30% of this threshold power. Spot size was set to 200 µ, pulse duration was 15 msec, and spot spacing was set to 0.25 spot diameter. A 2 × 2 pattern was used during the application. Laser application was repeated at three-month intervals during monthly examinations in cases where there was no complete recovery or there were recurrences.

A 512 × 128 macular cube scan was performed on all participants, and the quantitative evaluations included central macular thickness (CMT), which was automatically calculated using the Zeiss FORUM software. The automated measurement of CMT is obtained through macular cube scan acquisitions. Proprietary software algorithms subsequently compute the mean retinal thickness specifically within the central 1-millimeter diameter circular zone. This designated area directly aligns with the central subfield as defined by the Early Treatment Diabetic Retinopathy Study (ETDRS) macular grid. During subsequent follow-up visits, the auto-repeat and auto-alignment functionalities were employed to mitigate potential inaccuracies in intra-individual comparisons.

Statistical analysis was performed using SPSS, version 26.0, for Windows (IBM Corporation, Armonk, NY). For statistical analysis, the visual acuity data were converted to the logarithm of the minimum angle of resolution (logMAR). The Wilcoxon signed-rank test was performed to assess the change in macular thickness compared with the baseline. Statistical significance was set at *p* < 0.05.

## Results

Eleven eyes of 11 patients were included in the study. One patient was female, and ten were male. The ages of the patients were between 28 and 60 (mean 45.7 ± 8.4) years. The threshold laser power range was between 100 and 150 mW, with a mean of 133 ± 19 mW. The mean number of spots for each session of treatment was 450 ± 161.

**Fig. 2:**
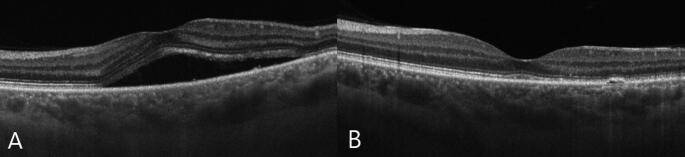
OCT image of a patient with CSR (**A**), post-laser resolution of subretinal fluid in the same patient (**B**)

**Fig. 3:**
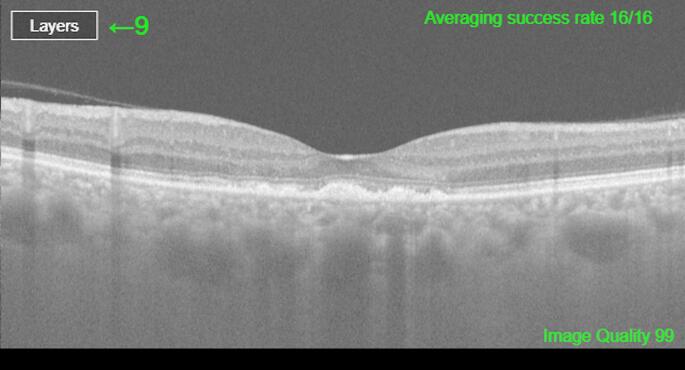
Retinal pigment epithelium (RPE) changes, specifically subfoveal RPE hypertrophy and mottling

The central macular thickness range measured by the pretreatment OCT was 268–432 μm. BCVA (logMAR) ranged between 0.69 and 0.17. Four (36.3%) patients had complete disappearance of the subretinal fluid with a single laser application and had no recurrence (Fig. 2). In seven (63.6%) patients, additional laser treatment was required due to the persistence of subretinal fluid. The treatment was applied two times for five (45.4%) patients, three times for one patient (9.1%), and five times for one patient (9.1%). In all patients with a one-year period, the absence of intraretinal or subretinal fluid on optical coherence tomography was achieved after the last treatment. The mean pre-treatment CMT value was 343.3 ± 50.2 μm, which decreased to 233.1 ± 20.3 μm following the treatment (*p* = 0.008). The posttreatment central macular thickness range was 194–270 μm. The mean BCVA (logMAR) values of patients prior to treatment were 0.46 ± 0.18, which significantly improved to a mean BCVA of 0.34 ± 0.31 with the treatment (*p* = 0.012). When patients were categorized into two distinct groups based on the number of laser treatments received (single versus multiple), no statistically significant difference in CMT was observed between the groups (*p* = 0.493, 343.7 ± 58.4 and 343.14 ± 44.8 μm, respectively). While eight (72.7%) patients had increased vision, two (18.1%) patients had no change in vision and one (9.1%) patient had decreased vision. The patient, who had decreased vision, received two laser treatments. The laser treatment was applied five times to one of the patients whose vision did not change and two times to the other one. Pigmentary alterations in the retinal pigment epithelium were observed in five patients (45.4%), encompassing manifestations such as RPE thickening, skip lesions, aggregation, atrophy, and mottling (Fig. 3). None of the patients developed retinal pigment epithelium atrophy, choroidal neovascularization, or any other retinal or choroidal complications (Table 1).

**Table 1:** Demographic and clinical characteristics of the patients

Patient no	Eye	Age	Gender	BCVA Pretreat-ment (logMAR)	BCVA Posttreat-ment (logMAR)	Macular Thickness Pretreat-ment (µm)	Macular Thickness Posttreat-ment (µm)	No of treatment	Complications
1	Left	28	M	0.47	0.39	416	194	2	RPE change
2	Right	54	F	0.47	0.30	268	216	1	RPE change
3	Right	46	M	0.17	0	318	270	2	
4	Left	46	M	0.69	1	301	209	2	RPE change
5	Right	41	M	0.47	0.30	333	241	1	
6	Left	47	M	0.39	0.30	342	226	1	
7	Right	44	M	0.39	0.30	432	232	1	
8	Right	40	M	0.90	0.90	357	234	5	RPE change
9	Left	44	M	0.39	0	310	247	2	
10	Left	50	M	0.47	0	400	245	3	RPE change
11	Right	60	M	0.30	0.30	300	251	2	RPE change

## Dıscussıon

Conventional focal photocoagulation has the effect of sealing the leak by forming fibrotic scar tissue with a thermal effect on RPE at the leakage site, but severe complications may occur ^3^. Nondamaging retinal laser therapy can be a useful alternative with minimal adverse effects in CSR. The main principle here is to reduce the power and pulse duration of the laser, thus reducing the amount of heat generated by the laser effect on the retina. By reducing the amount of energy applied, it is aimed at maintaining the therapeutic effect and eliminating the risk of retinal damage. This therapeutic effect does not occur by coagulation and scarring as in conventional laser treatment, but by the formation of the biological response to restore the outer blood-retinal barrier [12]. Although the mechanism of this biological response is not entirely clear, it has been claimed by some authors that the desired effect is achieved by activating the production of heat shock proteins (HSP) [11, 13, 14].

Heat shock proteins are expressed in response to a wide variety of conditions, such as hyperthermia, free oxygen radicals, ischemia, inflammation, and infection [15], [16] and they play a role in responding to stress and apoptosis. Stress-related functions of HSPs include the refolding, translocation, and degradation of proteins, thus acting as chaperones to maintain cytoskeletal integrity and metabolic homeostasis of cells [14]. They reduce inflammation and inhibit apoptosis [14]. Laser energy is absorbed by the retinal pigment epithelium and choroid, and it is converted into heat. The laser applied at the subthreshold level prevents heat energy from spreading to the photoreceptor layer and more inner retinal layers but creates the thermal effect that causes HSP expression. This increased synthesis and co-chaperones in RPE in response to the laser-induced thermal effect allows aging and damaged cells to return to their normal physiology, thereby increasing the ability to pump fluid from the retina to the choroid and decrease the choroidal permeability. Furthermore, there is no damage to the photoreceptor and inner retina layers due to the thermal effect [11].

Lavisky et al. analyzed the HSP70 expression by examining immunohistochemical confocal microscopy images of the laser spots in the RPE. At a 20% energy level, there was no expression in the laser spots, whereas at a 25% energy level, 57% of the laser spots showed expression. The expression was 98% when the energy level was 30% and 99% when the energy level rose to 40% [11].

While non-damaging laser treatments are relatively new, they are increasingly used in CSR treatment, especially in chronic cases. However, there is no standard in the application of this treatment in literature. Firstly, there is no consensus on the definition of chronic CSR. Yadav and Koss accepted cases lasting longer than three months as chronic, while Lavinsky and Chen accepted cases lasting longer than four months as chronic. Also, Kim and Ricci accepted cases lasting longer than six months as chronic [8]. In our study, we accepted cases lasting more than four months as chronic.

Yadav et al. applied a subthreshold micropulse (577 nm) yellow laser to the eyes of 13 patients with chronic CSR. They reported that the pre-treatment subretinal fluid height of the patients decreased from 232 to 49 μm post-treatment. Two of these 15 eyes had two lines of improvement, and four eyes had one line of improvement, while nine eyes did not have vision improvement [3]. Chen et al. applied a subthreshold micropulse 810 nm diode laser to the eyes of 25 patients with chronic CSR and followed them for six months. While more than half of the patients had decreased foveal thickness, 25% of the patients had no response after the laser in their study. They reported that the number of eyes with visual gain of three lines or more was 15 (57.7%), whereas the number of eyes with gain between one and three lines was six (23.1%) [17]. Jeffery et al. also applied subthreshold micropulse 810 nm diode lasers to the eyes of 11 patients. Six of these 11 eyes had acute CSR, and five of them had chronic CSR. They found that the retinal thickness of the patients followed for an average of 14 months decreased from 314 to 893 μm (mean 508 μm) pretreatment to 222–365 μm (mean 250 μm) posttreatment. Subretinal fluid disappeared in all patients after treatment, but only one eye required re-treatment in their study [7]. Jeffery et al. stated that a high amount of laser spot application could be effective in achieving these high success rates (about 772 spots for each treatment). However, this high success may also be due to the fact that more than half of the patients already have acute CSR with a high probability of spontaneous recovery. Lavisky et al. applied a 577 nm PASCAL laser with EpM to 16 eyes with chronic CSR and found that mean central macular thickness decreased from 350 μm to 282 μm. In 75% of these patients, subretinal fluid completely resolved at the end of the sixth month, while minimal fluid remained in 25% of the patients [10]. In our study, we used the same system as Lavinsky et al. and observed that subretinal fluid resolved in all patients; the pretreatment maximum retinal thickness ranged between 268 and 432 μm, and the posttreatment maximum retinal thickness ranged between 194 and 270 μm.

Roisman et al. compared subthreshold micropulse laser with the sham group, and they reported that the vision improved by more than 15 letters in the laser group [18]. Similarly, in our study, there was an increase in vision in eight patients and a decrease in vision in two patients.

In most studies, the rate of patients that required treatments more than once was clustered between 42% and 80% (8). Lanzetta reported that in 42% of patients, more than once treatment was required, while Lavinsky reported that it was required in 84% of patients [1118]. Other researchers, such as Jeffery et al., have reported a low rate of retreatment [7]. In our study, seven (63.6%) of 11 patients required retreatment. The laser treatment was applied two times to five patients (45.4%); one patient (9.1%) required three laser applications; and one patient (9.1%) required five laser treatments.

Lanzetta, Roisman, Chen, Ricci, and Koss used subthreshold micropulse 810 nm diode lasers, and Kim, Yadav, Scholz, and Lavinsky used subthreshold micropulse 577 nm yellow lasers in the treatment of central serous chorioretinopathy [12, 17–22]. None of the authors found any complications during examination or OCT and FFA imaging except Lanzetta. Lanzetta reported pigmentary changes in the RPE in some of the patients [19]. In our study, five of 11 patients had pigmentary changes in the RPE. Despite the fact that only a single laser treatment was administered to one of these five patients, changes in the RPE were observed. Based on this observation, we can hypothesize that the RPE changes might be a consequence of either the natural healing process of CSR or the laser treatment(s) we employed. Unfortunately, there is no standard in literature on this subject.

The low number of patients and the lack of a control group are the limitations of our study.

In conclusion, we found that the PASCAL device using the 577 nm yellow laser was effective and reliable in the management of chronic CSR in this study. We obtained a one-year follow-up period without subretinal fluid in all our patients. We did not encounter any complications except pigmentary changes in the RPE. Although the standardization issues are still unresolved, subthreshold laser treatment using PASCAL with EpM has improved the management of chronic CSR. Further studies are needed to establish the standards for subtreshold laser application.

## Data Availability

No datasets were generated or analysed during the current study.
